# Bmi-1 overexpression as an efficient prognostic marker in patients with nonsmall cell lung cancer

**DOI:** 10.1097/MD.0000000000007346

**Published:** 2017-06-30

**Authors:** Xiaojun Zhang, Tian Tian, Wei Sun, Changting Liu, Xiangqun Fang

**Affiliations:** Nanlou Respiratory Diseases Department, Chinese PLA General Hospital, Beijing, China.

**Keywords:** Bmi-1, lung cancer, meta-analysis, prognosis, survival

## Abstract

**Background:**

The prognostic effect of B-cell-specific Moloney leukemia virus insertion site 1 (Bmi-1) in patients with nonsmall cell lung cancer (NSCLC) remains controversial. We thus performed a meta-analysis to reveal the correlation between Bmi-1 with clinical features and overall survival (OS) in NSCLC.

**Methods:**

Relevant studies were searched through PubMed, Embase, and Web of Science. Pooled hazard ratios (HRs) and 95% confidence intervals (CIs) as well as odds ratios (ORs) and 95% CIs were calculated by using STATA version 12.0.

**Results:**

Fourteen studies consisting of 1323 patients were included for quantitative analysis. The results showed that Bmi-1 was significantly associated with tumor size (n = 7, OR = 1.79, 95% CI = 1.19–2.71, *P* = .005, fixed effect), poor differentiation (OR = 1.61, 95% CI = 1.11–2.33, *P* = .011, fixed effect), and distant metastasis (n = 4, OR = 4.69, 95% CI = 1.52–14.41, *P* = .007, fixed effect). In addition, high Bmi-1 expression also predicted poor OS (HR = 1.62, 95% CI = 1.14–2.3, *P* < .001). There was no significant publication bias for any of the analyses.

**Conclusion:**

In conclusion, Bmi-1 overexpression was correlated with tumor size, poor differentiation, distant metastasis, and worse OS in NSCLC. Therefore, Bmi-1 could be recommended as an efficient prognostic marker for NSCLC.

## 1 Introduction

Lung cancer remains the most common cancer among all cancer types worldwide.^[1]^ Nonsmall cell lung cancer (NSCLC) accounts for approximately 85% of all lung cancer cases and is often asymptomatic at early stages.^[2]^ In the past several decades, therapeutic approaches for NSCLC have undergone considerable progress. In addition to traditional therapies such as surgical resection, chemotherapy, and radiotherapy, new strategies such as targeted therapy and immunotherapy were also introduced.^[3–5]^ Faced with various therapeutic options, clinicians require reliable biomarkers to devise the optimal regimens. Furthermore, for a given treatment that was prescribed according to a standard dosing protocol, different patients could have significantly diverse survival outcomes. Therefore, novel biomarkers are urgently required to facilitate personalization of NSCLC treatment.^[6]^

B-cell-specific Moloney leukemia virus insertion site 1 (Bmi-1) is a structural component of the polycomb repressive complex 1.^[7]^ Growing evidence has shown that Bmi-1 plays an important role in the self-renewal of cancer stem cells (CSCs).^[8,9]^ Bmi-1 overexpression could promote cell proliferation and induce leukemia initiation through the *ink4a* locus.^[10]^ Bmi-1 was also involved in the pathogenesis of medulloblastomas by activation of the sonic hedgehog pathway.^[11]^ Furthermore, Bmi-1 was required for self-renewal activity and stemness-maintenance in prostate cancer.^[12]^ Recent studies demonstrated that Bmi-1 participated in the occurrence of epithelial–mesenchymal transition (EMT) by repressing both E-cadherin and p16INK4a and further contributed to tumor development in head and neck cancer.^[13]^ Moreover, Bmi-1 could repress the tumor suppressor gene PTEN to induce EMT.^[14]^ Current evidence suggests that the promotive role of Bmi-1 in tumorigenesis and Bmi-1 was observed to be upregulated in a variety of tumors including esophageal adenocarcinoma,^[15]^ breast cancer,^[16]^ colorectal cancer,^[17]^ gastric cancer,^[18]^ and NSCLC.^[19–22]^ However, regarding the prognostic value of Bmi-1 in NSCLC, different research groups^[20–23]^ presented controversial results. Therefore, we carried out a meta-analysis by collecting and pooling the most relevant and recent studies to obtain statistical evidence to reveal the significance of Bmi-1 for prognostication in NSCLC.

## 2 Materials and methods

### 2.1 Search strategy

We conducted a comprehensive literature search through the electronic platforms of PubMed, Embase, and Web of Science until April 2017. The search strategy included terms regarding “Bmi-1” (“B-cell-specific Moloney leukemia virus insertion site 1” or “Bmi-1” or “bmi1”) and “non-small cell lung cancer” (“lung neoplasms” [MeSH Terms] or “lung cancer” or “lung tumor” or “lung carcinoma” or “NSCLC”). Reference lists of retrieved articles were reviewed to find additional studies. This meta-analysis was performed in accordance with the Preferred Reporting Items for Systematic Reviews and Meta-Analyses statement.^[24]^ Since this is a meta-analysis, no ethics committee or institutional review board approval was necessary for the study.

### 2.2 Inclusion and exclusion criteria

Studies fulfilling the following requirements were considered as eligible: the histological type of disease was NSCLC and was pathologically confirmed; the expression of Bmi-1 was determined by real-time reverse transcriptase polymerase chain reaction or immunohistochemistry; studies investigated the relationship between Bmi-1 and clinical factors or overall survival (OS) in NSCLC; hazard ratios (HRs) and 95% confidence intervals (CIs) or odds ratios (ORs) and 95% CIs were given in the text or could be computed by Tierney formulas^[25]^; and full-text English publications. The exclusion criteria were nonfull-text papers, reviews, and case reports; animal studies; and duplicate or overlapped studies.

### 2.3 Data extraction

Two investigators (XZ and TT) independently extracted the following data from eligible studies: first author's name, year of publication, study country, number of patients, survival outcomes, tumor stage, methods for Bmi-1 detection, and HRs and 95% CIs. Any disagreement between the 2 investigators was settled by consultation with the third investigator (XF).

### 2.4 Statistical analysis

ORs with 95% CIs were selected to assess the correlation between Bmi-1 and clinical characteristics of patients. HRs and 95% CIs were utilized to explore the impact of Bmi-1 on OS. Heterogeneity among studies was evaluated by using Cochran Q test and the Higgins I^2^ statistic. I^2^>50% or *P*_heterogeneity_ < .10 indicated significant heterogeneity, in which case a random-effects model was used; otherwise, a fixed-effects model was applied. To detect the potential publication bias, both Begg test and Egger test were used. All analyses were conducted by using STATA version 12.0 (Stata Corp., College Station, TX). *P* value <.05 was considered as statistically significant.

## 3 Results

### 3.1 Study selection and characteristics of included studies

**Figure 1:**
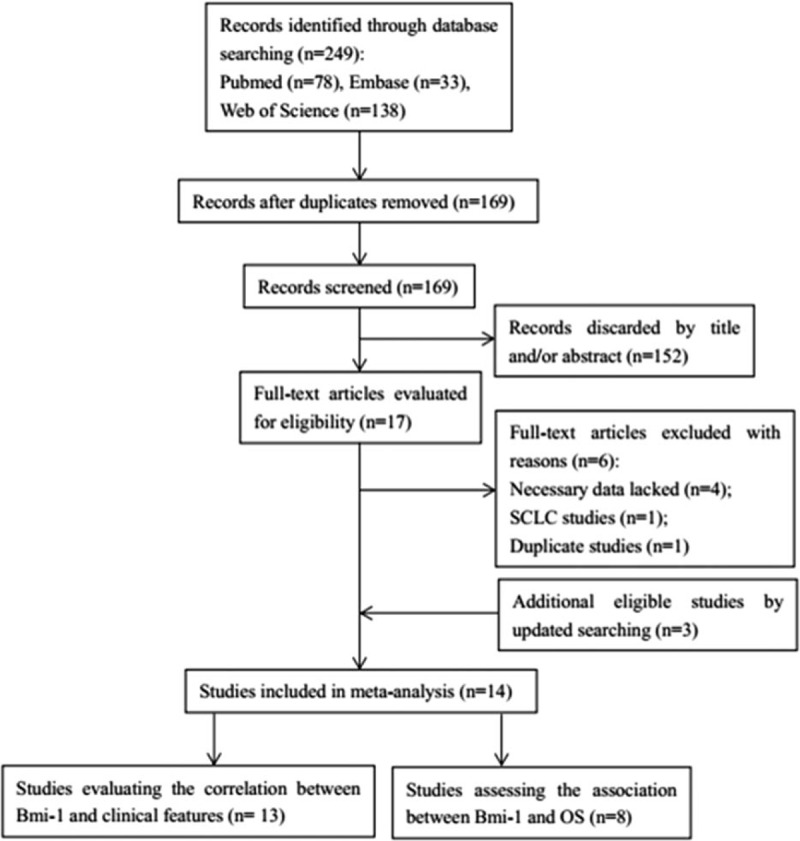
Flow chart of study selection.

The initial literature search identified 249 potentially relevant studies. As illustrated in Figure 1, 169 records remained after duplicates were removed and they were reviewed by checking the title and abstract. Subsequently, 152 articles were eliminated because they were irrelevant studies, reviews, conference abstracts, or nonhuman studies. Seventeen full-text studies were further evaluated and 6 records were excluded because 4 studies lacked adequate data, 1 study was on patients with small cell lung cell, and 1 study was based on the same patient group as another study. Three additional eligible studies were identified by updated searching. Finally, 14 studies^[19–23,26–34]^ consisting of 1323 patients were included for quantitative analysis. Thirteen studies^[19–23,26–29,31–34]^ reported the association between Bmi-1 and clinical factors in NSCLC and 8 studies^[22,27,29–34]^ investigated the correlation between Bmi-1 and OS. The studies were published from 2001 to 2017 and were from 5 countries: China, Japan, Italy, Greece, and Switzerland. Eleven studies^[21–23,26–29,31–34]^ were performed on Asian patients and 3 studies^[19,20,30]^ recruited Caucasian patients. All patients included in each eligible study belonged to the same race. The sample sizes ranged from 30 to 199, with a median of 80. Major characteristics are summarized in Table 1.

**Table 1:**
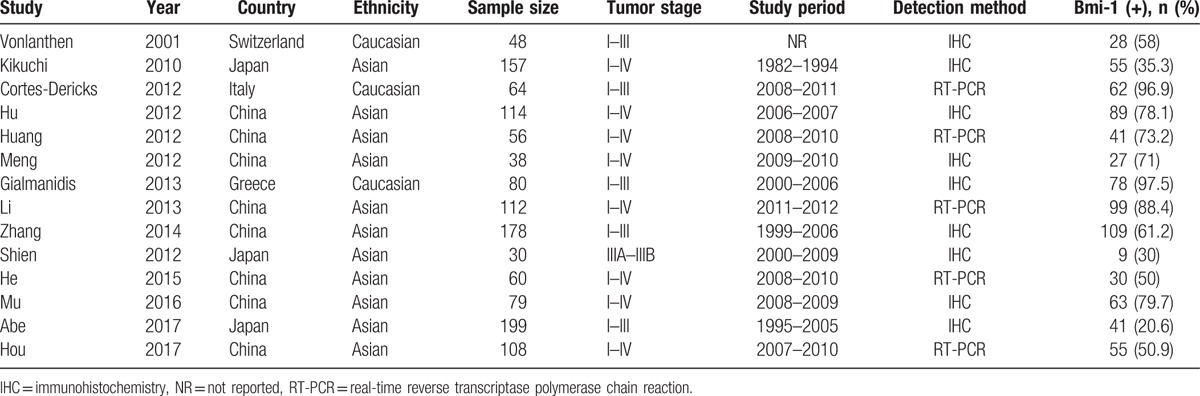
Main characteristics of all included studies.

### 3.2 Correlation of Bmi-1 expression with clinical parameters

We extracted data to establish the association of Bmi-1 with 9 clinical factors for a meta-analysis. These clinical characteristics were age, gender, tumor/node/metastasis (TNM) stage, histological type, lymph node metastasis, tumor size, differentiation, smoking status, and distant metastasis. As shown in Table 2, there was significant association between Bmi-1 expression and tumor size (n = 7, OR = 1.79, 95% CI = 1.19–2.71, *P* = .005, fixed effect). Pooled data from 7 studies also showed that Bmi-1 was associated with poor differentiation (OR = 1.61, 95% CI = 1.11–2.33, *P* = .011, fixed effect, Table 2). In addition, Bmi-1 overexpression was found to be positively correlated with distant metastasis (n = 4, OR = 4.69, 95% CI = 1.52–14.41, *P* = .007, fixed effect, Table 2). By contrast, the combined results demonstrated no association between Bmi-1 and age, gender, TNM stage, lymph node metastasis, histology, or smoking status (Table 2). These findings suggested that high Bmi-1 expression was an indicator of aggressive biological behavior and distant dissemination of the disease.

**Table 2:**
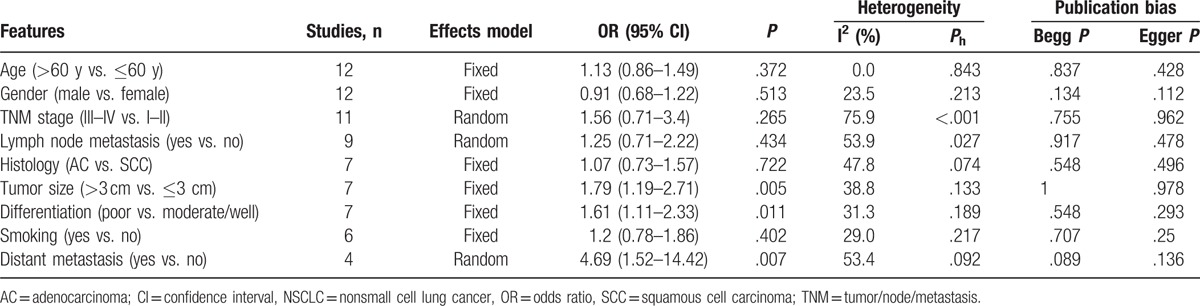
Relationship between Bmi-1 and clinical features in NSCLC.

### 3.3 Association of Bmi-1 expression with OS

**Figure 2:**
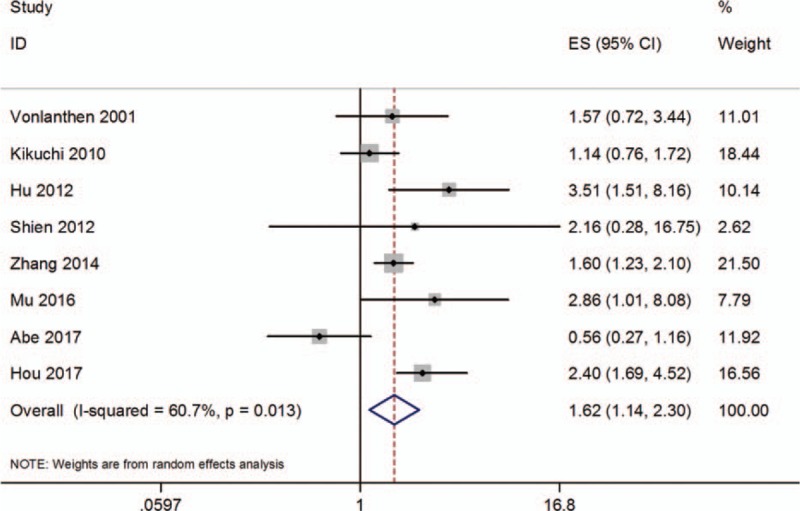
Forest plot of correlation between Bmi-1 with overall survival in nonsmall cell lung cancer.

Eight studies^[22,27,29–34]^ with 913 subjects explored the influence of Bmi-1 on OS in patients with NSCLC. As significant heterogeneity among studies was detected, a random-effects model was adopted. The pooled HR and 95% CI for OS was 1.62 and 1.14 to 2.3, respectively (*P* < .001, Fig. 2). The results indicated that Bmi-1 was predictive for shorter OS in the patients with NSCLC.

### 3.4 Publication bias

**Figure 3:**
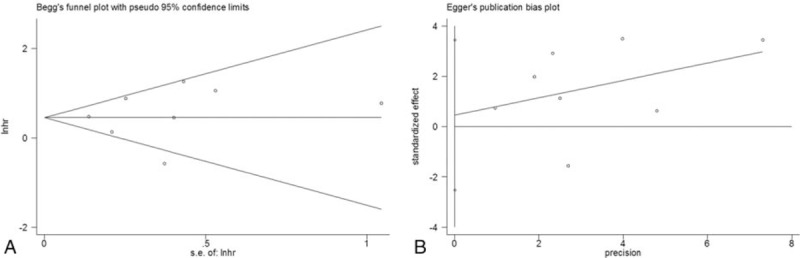
Publication bias tested by (A) Begg funnel plot (*P* = .536) and (B) Egger test (*P* = .37) for the association between Bmi-1 and overall survival.

Begg funnel plot and Egger linear regression test were simultaneously used to analyze publication bias. As shown in Table 2 and Figure 3, all *P* values for publication bias were >.05, confirming that there was no publication bias in the present study.

## 4 Discussion

Recent studies have reported various biomarkers for therapeutic effects and estimation of survival outcomes in NSCLC,^[6,35,36]^ of which Bmi-1 has attracted extensive attention. A large number of studies^[19–23,26–31]^ explored the utilization of Bmi-1 for prognosis; however, the results were inconsistent. Considering that the small sample size may attenuate the objectivity of the results, we gathered data regarding Bmi-1 expression in NSCLC from 14 studies for analysis. The results demonstrated a significant association between Bmi-1 expression and large tumor size, poor differentiation, and distant metastasis in NSCLC. In addition, high Bmi-1 expression also could predict worse OS in patients with NSCLC. To our knowledge, this was the first study exploring the impact of Bmi-1 on clinical variables and survival outcomes in NSCLC by a meta-analysis.

Bmi-1 was first found to be involved in the generation of mouse lymphomas in 1991.^[37,38]^ Subsequent studies showed that Bmi-1 also played an essential role in the self-renewal of hematopoietic, neural, and intestinal stem cells.^[11,39,40]^ Bmi-1 could regulate the self-renewal property of tumorigenic human mammary stem cells, in cooperation with the hedgehog pathway.^[41]^ The capability of self-renewal and multilineage differentiation in tumorigenesis, which was activated and promoted by Bmi-1 expression, could be the potential mechanisms underlying the correlation between Bmi-1 and poor differentiation in NSCLC. Furthermore, Bmi-1 also participated in EMT during cancer development by the repression of tumor suppressor genes.^[14]^ EMT in cancer cells leads to a loss of contact inhibition, remodeling of extracellular matrix components, and activation of different growth factors. These processes could ultimately result in cancer cell migration and metastasis. The link between Bmi-1 and EMT could be a possible reason for the association between Bmi-1 and distant metastasis in this meta-analysis. Emerging evidence showed that CSCs exhibiting excessive Bmi-1 levels were increasingly resistant to chemotherapy.^[42–44]^ Collectively, these studies implied that Bmi-1 might be a potential therapeutic target for cancer.

Several studies^[45,46]^ also investigated the prognostic value of Bmi-1 in patients with cancer through a meta-analysis. Shao et al^[45]^ showed that Bmi-1 was a negative predictor for OS in Asian patients of various cancer types (HR = 1.96, 95% CI: 1.62–2.36). Yuan et al^[46]^ found that Bmi-1 was significantly associated with tumor size, T classification, lymph node metastasis, distant metastasis, as well as poor OS in gastric cancer. These results were in line with the findings of the present study. Moreover, we conducted a publication bias examination for all analyses using both Begg test and Egger test, and no significant publication bias was detected, which guaranteed the stability of our study.

Some limitations should be noted in the present meta-analysis. First, only 8 studies reporting the association between Bmi-1 and OS were included. Further subgroup analysis for OS could not be performed owing to the relatively small sample size. Second, of all 14 eligible studies, only 3 of them were conducted on Caucasian patients and the most patients were from Asian countries. The imbalance of patient ethnicities may restrict the general applicability of the results in Caucasian patients.

In summary, the present meta-analysis demonstrated that Bmi-1 overexpression was correlated with tumor size, differentiation, and distant metastasis in NSCLC. Meanwhile, Bmi-1 was also indicated as a biomarker for poor OS. To strengthen our conclusion, large-scale studies with diverse ethnical backgrounds are warranted.
